# Postoperative ctDNA could potentially spare patients from unnecessary adjuvant therapy in HPV-negative head and neck squamous cell carcinoma: a meta-analysis

**DOI:** 10.3389/fonc.2026.1902328

**Published:** 2026-10-16

**Authors:** Mohammad Rehan Asad, Alejandra Flores Pusarico, Nidhi Reji, Tareq Nayef AlRamadneh, Atta Ullah Khan

**Affiliations:** 1 Department of Basic Medical Science, Majmaah University, Al Majmaah, Saudi Arabia; 2 Facultad de Medicina, Universidad Mayor de San Andres, La Paz, Bolivia; 3 Department of General Surgery, The Rotherham National Health Service (NHS), Rotherham General Hospital, Rotherham, United Kingdom; 4 Faculty of Allied Medical Sciences, Hourani Center for Applied Scientific Research, Al-Ahliyya Amman University, Amman, Jordan; 5 Department of Precision Medicine, University of Campania “Luigi Vanvitelli”, Napoli, Italy

**Keywords:** circulating tumor DNA, head and neck cancer, HPV, prognosis, squamous cell carcinoma

## Abstract

**Background:**

The prognostic utility of circulating tumor DNA (ctDNA) for minimal residual disease detection in human papillomavirus (HPV)-negative head and neck squamous cell carcinoma (HNSCC) remains incompletely characterized. This meta-analysis evaluates the association between post-operative ctDNA and recurrence-free survival (RFS) in this population.

**Methods:**

A systematic search of major databases through May 2026 identified studies evaluating post-operative ctDNA and RFS in HPV-negative HNSCC. Pooled hazard ratios (HRs) were calculated using a random-effects model.

**Results:**

Eight studies (n=325) were included. Detectable post-operative ctDNA was associated with diminished RFS (HR = 10.07, 95% CI: 5.64–17.99) and overall survival (HR = 8.98, 95% CI: 2.93–27.58). However, sensitivity analyses substantially attenuated the pooled RFS estimate (HR range: 2.22–2.43), suggesting the primary effect magnitude may lack stability.

**Conclusions:**

While preliminary evidence suggests an association between post-operative ctDNA and recurrence risk in HPV-negative HNSCC, these findings require cautious interpretation. The current literature is constrained by small sample sizes, heterogeneous detection methodologies, mixed-cohort inclusion, and potential confounding biases. Consequently, post-operative ctDNA remains an investigational biomarker in this setting. Clinical implementation necessitates further validation through large, prospective, multi-institutional studies employing standardized methodologies in strictly defined HPV-negative #146;populations.

## Introduction

Head and neck squamous cell carcinoma (HNSCC) is the seventh most common malignancy worldwide, accounting for approximately 890, 000 new cases annually (1). While human papillomavirus (HPV) infection drives a subset of oropharyngeal cancers, the majority of HNSCC cases remain HPV-negative, predominantly associated with tobacco and alcohol consumption (2). HPV-negative HNSCC carries a poorer prognosis than HPV-positive disease, necessitating improved prognostic stratification and therapeutic monitoring (3).

Despite advances in multimodal treatment, disease recurrence occurs in 50-60% of patients following curative-intent treatment, with most relapses occurring within two years (1, 4). Current surveillance relies on clinical examinations and radiological imaging, which lack sensitivity for detecting microscopic residual disease (5). Molecular residual disease (MRD) refers to the detection of tumour-derived DNA after curative-intent treatment and before clinically or radiologically confirmed recurrence. Post-treatment ctDNA may therefore function as a marker of MRD; however, studies differ in whether MRD detection was their explicit clinical application or whether ctDNA was assessed more broadly for recurrence monitoring, ctDNA clearance, or surveillance. Therefore, reliable non-invasive biomarkers capable of detecting minimal residual disease (MRD) and predicting recurrence-free survival (RFS) are critically needed to guide adjuvant therapy decisions (6).

Circulating tumor DNA (ctDNA) has emerged as a promising liquid biopsy biomarker in precision oncology. While ctDNA utility is well-established in HPV-positive HNSCC, its application in HPV-negative disease presents unique challenges due to genetic heterogeneity and high mutational burden (7). Recent advances in tumor-informed personalized ctDNA assays using next-generation sequencing (NGS) and digital droplet PCR (ddPCR) have enhanced sensitivity and specificity in non-viral solid tumors (8). Tumor-informed assays demonstrate superior performance compared to tumor-agnostic approaches, with reported sensitivities exceeding 90% and specificities approaching 99% (9). Emerging evidence demonstrates that post-operative ctDNA positivity predicts disease recurrence and inferior survival outcomes in colorectal, breast, and lung cancers (10, 11), with preliminary studies suggesting similar prognostic value in HPV-negative HNSCC (7). The LIONESS trial demonstrated feasibility of personalized ctDNA analysis for MRD detection in HNSCC.

However, the prognostic significance of post-operative ctDNA in HPV-negative HNSCC remains incompletely characterized, with existing literature comprising small, heterogeneous cohorts using varying methodologies. This systematic review and meta-analysis synthesizes available evidence on the prognostic value of post-operative ctDNA for predicting RFS in HPV-negative HNSCC following curative-intent treatment.

## Methods

### Study design and registration

This systematic review and meta-analysis adheres to the Preferred Reporting Items for Systematic Reviews and Meta-Analyses (PRISMA) 2020 guidelines (12). The protocol was prospectively registered in PROSPERO (CRD420261390645). The primary objective was to evaluate the prognostic significance of post-operative ctDNA detection on RFS in HPV-negative HNSCC patients undergoing curative-intent treatment.

### Search strategy

A comprehensive literature search was conducted across PubMed/MEDLINE, Embase, Cochrane CENTRAL, and Web of Science from database inception through 8^th^ May 2026. The search strategy combined Medical Subject Headings and free-text keywords: (“head and neck squamous cell carcinoma” OR “HNSCC” OR “head and neck cancer”) AND (“circulating tumor DNA” OR “ctDNA” OR “cell-free DNA” OR “liquid biopsy” OR “minimal residual disease” OR “MRD”) AND (“prognosis” OR “survival” OR “recurrence” OR “relapse”). Reference lists of included articles and conference abstracts from ASCO and ESMO were manually reviewed to identify additional eligible studies. For detailed search strategy please refer to **Supplementary Data Table 1**.

### Eligibility criteria

Studies were included if they met the following PICOS criteria: (1) Population: Adult patients (≥18 years) with HPV-negative HNSCC (oral cavity, larynx, hypopharynx, HPV-negative oropharynx) or mixed population studies with predominantly HPV Negative population undergoing curative-intent treatment; (2) Intervention: Post-operative ctDNA detection in plasma or serum; (3) Comparator: Undetectable post-operative ctDNA; (4) Outcomes: Recurrence-free survival (primary), overall survival (5) Study Design: Prospective/retrospective cohort, case-control, or randomized controlled trials. Studies reporting hazard ratios (HRs) with 95% confidence intervals (CIs) or sufficient Kaplan-Meier data were included.

Exclusion criteria included HPV-positive disease, nasopharyngeal carcinoma, non-squamous histologies, case reports, reviews, non-English publications, and studies lacking survival data.

### Study selection and data extraction

Two independent reviewers screened titles and abstracts, then evaluated full texts against inclusion criteria. Disagreements were resolved by consensus or third-party adjudication. Data extraction included study characteristics (author, year, country, design), patient demographics (sample size, age, sex, tumor site, stage), treatment details, ctDNA methodology (assay type, platform, timing), and clinical outcomes (HRs with 95% CIs, follow-up duration, recurrence rates). When HRs were not reported, they were estimated from Kaplan-Meier curves using Tierney et al. methods (13).

### Quality assessment and risk of bias

Two reviewers independently will assess study quality using the Quality In Prognosis Studies (QUIPS) tool (14). Publication bias will be evaluated using funnel plots, Egger’s linear regression test, and Begg’s rank correlation test, with p-values <0.05 indicating significant asymmetry (15, 16). Given the anticipated number of studies, formal publication bias assessment will be performed if ≥10 studies are included in the meta-analysis; otherwise, visual inspection of funnel plots will be conducted with narrative discussion of potential bias.

### Statistical analysis

Meta-analyses were performed using Review Manager 5.4. The primary effect measure was the hazard ratio (HR) for RFS comparing detectable versus undetectable post-operative ctDNA. Pooled HRs and 95% CIs were calculated using the inverse variance method with a random-effects model (DerSimonian-Laird) (17).

Statistical heterogeneity was assessed using Cochran’s Q test (p < 0.10) and I^2^ statistic, with I^2^ >75% indicating high heterogeneity. Predefined subgroup analyses examined ctDNA methodology (tumor-informed vs. tumor-agnostic), timing of blood sampling, and tumor stage. Sensitivity analyses sequentially omitted individual studies. Publication bias was assessed using funnel plots and Egger’s/Begg’s tests (if ≥10 studies included). Statistical significance was set at p < 0.05.

## Results

### Literature synthesis

Systematic searching yielded 4, 694 records, with 117 progressing to full-text evaluation. Following rigorous application of predefined inclusion criteria, eight studies were deemed eligible for qualitative synthesis and subsequent meta-analytic aggregation (**Figure 1**) (18–25). The attrition from initial identification to final inclusion reflected the stringent methodological requirements imposed on prognostic biomarker investigations, particularly regarding outcome reporting and population specificity.

**Figure 1:**
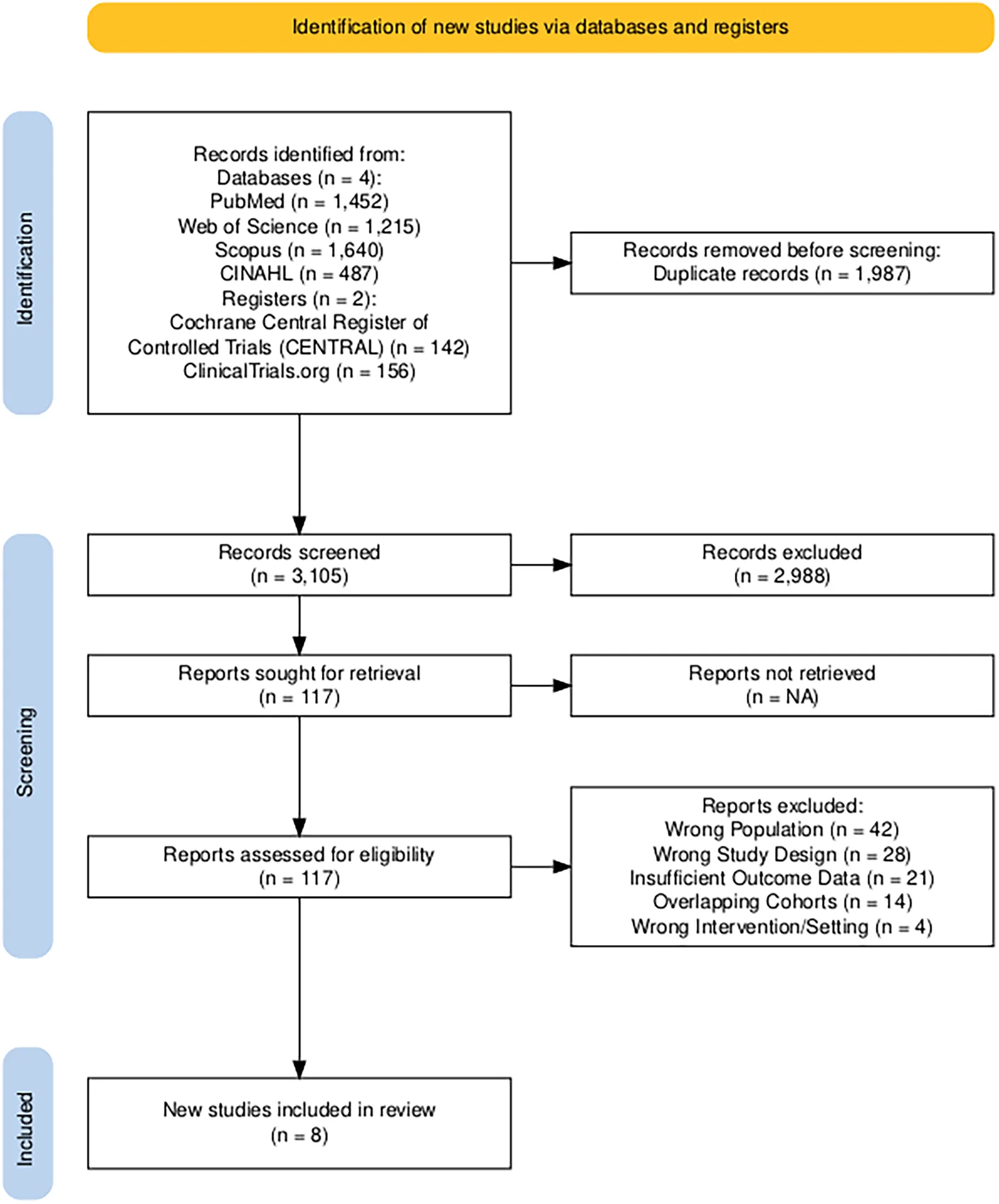
PRISMA Flow chart for study selection.

### Study population and methodological heterogeneity

The included cohort comprised 325 participants across five prospective and three retrospective investigations, with substantial representation of HPV-negative disease (approximately 55%). Considerable heterogeneity characterized the ctDNA detection architectures employed, encompassing NGS-based platforms, commercial tumor-informed assays, and emerging pooled methodologies (**Table 1**). This technological diversity reflects the nascent and rapidly evolving nature of liquid biopsy implementation in head and neck oncology. The temporal relationship between surgical intervention and biomarker assessment ranged considerably, from immediate post-operative periods to delayed sampling intervals, potentially introducing variability in ctDNA kinetics and detection concordance. Outcome ascertainment protocols demonstrated consistency in primary endpoint definitions, though institutional variation in surveillance methodology may have influenced recurrence detection sensitivity.

**Table 1:** Baseline characteristics of included studies.

Author	Study design	Sample size	Cohort type (HPV/p16 status)	Cancer sites	Cancer stage	Plasma collection timepoint	ctDNA test	Follow-up (months)	Outcome reported
Wu, (24)	Prospective	27	HPV-16-negative	Oral cavity, hypopharynx, larynx; distribution NR	I–IV; distribution NR	1–2 weeks after surgery	Targeted-capture NGS, 1021-gene panel	20.7, median (range 1.3–27.2)	DFS
Burgener, (23)	Prospective observational	30	HPV-negative	NR in supplied main text	I–IVA	At diagnosis and 3 months after surgery; additional samples before/during radiotherapy and/or at 12 months where applicable	Tumour-naïve CAPP-seq + cfMeDIP-seq	41.5, median	RFS
Lele, (25)	Retrospective	29	Mixed; 7 p16-positive oropharyngeal cancers; remaining HPV/p16 detail incompletely reported	Oral cavity (6), oropharynx (10), larynx (12), hypopharynx (1)	I/II (5), III/IV (23), NR (1)	8–12 weeks after treatment; then every 3 months	Personalised tumour-informed 16-plex mPCR-NGS (Signatera)	16, median (range 6–43)	RFS, OS
Honoré, (18)	Retrospective analysis of a prospectively enrolled biomarker cohort	43; 42 post-treatment evaluable	p16-defined mixed cohort: 17 p16-positive and 26 p16-negative; direct HPV status NR	Oropharynx (27), larynx (6), hypopharynx (5), oral cavity (5)	p16-negative sites: III/IVa/IVb; p16-positive oropharynx: I/II/III	Within 12 weeks after completion of curative-intent treatment	Personalised tumour-informed WES-guided assay (Signatera RUO; up to 16 SNVs)	16, median (range 1–42)	RFS, OS
Sim, (19)	Prospective	24	Predominantly HPV-independent (23/24 in abstract); formal HPV testing: 13 negative, 1 positive, 10 not tested	Oral cavity, larynx/hypopharynx, oropharynx	II–IVB	Serial early postoperative samples, 1–9 days; first sample median 1 day (range 1–3), plus surveillance	MAESTRO-Pool, ultrasensitive tumour-informed whole-genome assay	22.5, median (IQR 18.6–24.8)	RFS, OS
Karasik, (20)	Observational; design not stated in conference abstract	57	HPV-negative	Oral cavity (40/57); other sites NR	I–IVB	Postoperative, before any adjuvant therapy; exact interval NR	Personalised tumour-informed Signatera assay	11.8, median (range 1.7–23.4)	RFS
Ruiz-Torres, (22)	Prospective cohort	40	38 HPV-independent; 2 HPV-associated	Oral cavity, oropharynx, larynx, hypopharynx	I/II (5); III/IVA/IVB (35)	Before surgery when feasible; early postoperative; after treatment completion; surveillance	Tumour-informed PredicineBEACON MRD assay	33.7, median (range 7.1–40.4)	RFS, OS
Flach, (21)	Prospective, single-centre non-interventional cohort	76 analytic; 83 enrolled	p16-negative (71); p16-positive (5); direct HPV status NR	Oral cavity, pharynx, larynx	I–IVB	1–4 days preoperative; 2–7 days postoperative; serial follow-up	Personalised tumour-informed RaDaR assay (WES-informed)	29.5, median (range 0–57)	RFS

ctDNA, circulating tumour DNA; DFS, disease-free survival; EFS, event-free survival; HPV, human papillomavirus; mPCR-NGS, multiplex polymerase chain reaction–next-generation sequencing; MRD, minimal residual disease; NR, not reported.

### MRD-focused studies and clinical performance

Five of the eight included studies used MRD or residual-disease terminology, or framed post-treatment ctDNA detection as an MRD application. These were the studies by Honoré, Sim, Karasik, Ruiz-Torres, and Flach (18–22). The remaining studies evaluated postoperative or post-treatment ctDNA primarily in the contexts of relapse monitoring, ctDNA clearance, or PET-adjunct surveillance (23–25).

Clinical performance among the MRD-focused studies was encouraging but heterogeneous. In the study by Honoré et al., ctDNA was detected in 4 of 42 evaluable patients within 12 weeks after curative-intent treatment; three of these patients relapsed, and the median lead time before clinical recurrence was 7.0 months (range, 3.6–7.1 months) (18). Sim et al. reported a positive predictive value of 92.9% and a negative predictive value of 80% for immediate postoperative ctDNA detection in relation to recurrence or death; the first postoperative sample had 86.7% sensitivity and 88.9% specificity (19). Karasik et al. reported lower actuarial one-year recurrence-free survival among ctDNA-positive than ctDNA-negative patients (38.2% vs 87.4%) (20). Ruiz-Torres et al. reported patient-level sensitivity and specificity of 78% and 91%, respectively, during the first six weeks after treatment completion, and 89% and 87%, respectively, during subsequent surveillance (22). In the LIONESS cohort, Flach et al. reported 91.3% sensitivity, 94.2% specificity, 87.5% positive predictive value, and 96.1% negative predictive value for recurrence detection using plasma samples collected at least 14 days after surgery (21). A study-level summary of MRD framing and the reported clinical performance of post-treatment ctDNA testing is provided in **Supplementary Table S2**.

### Quality appraisal and bias considerations

QUIPS-based assessment revealed that the majority of included studies demonstrated low risk of bias across most evaluated domains. However, approximately one-third of investigations exhibited substantive confounding bias, suggesting potential residual confounding that warrants cautious interpretation of effect estimates (**Figure 2**). The predominant strength across domains of study participation, attrition, measurement validity, and analytical rigor provides some reassurance regarding the internal validity of the aggregated evidence, though the heterogeneous confounding profile necessitates sensitivity analyses and subgroup stratification in subsequent meta-analytic procedures.

**Figure 2:**
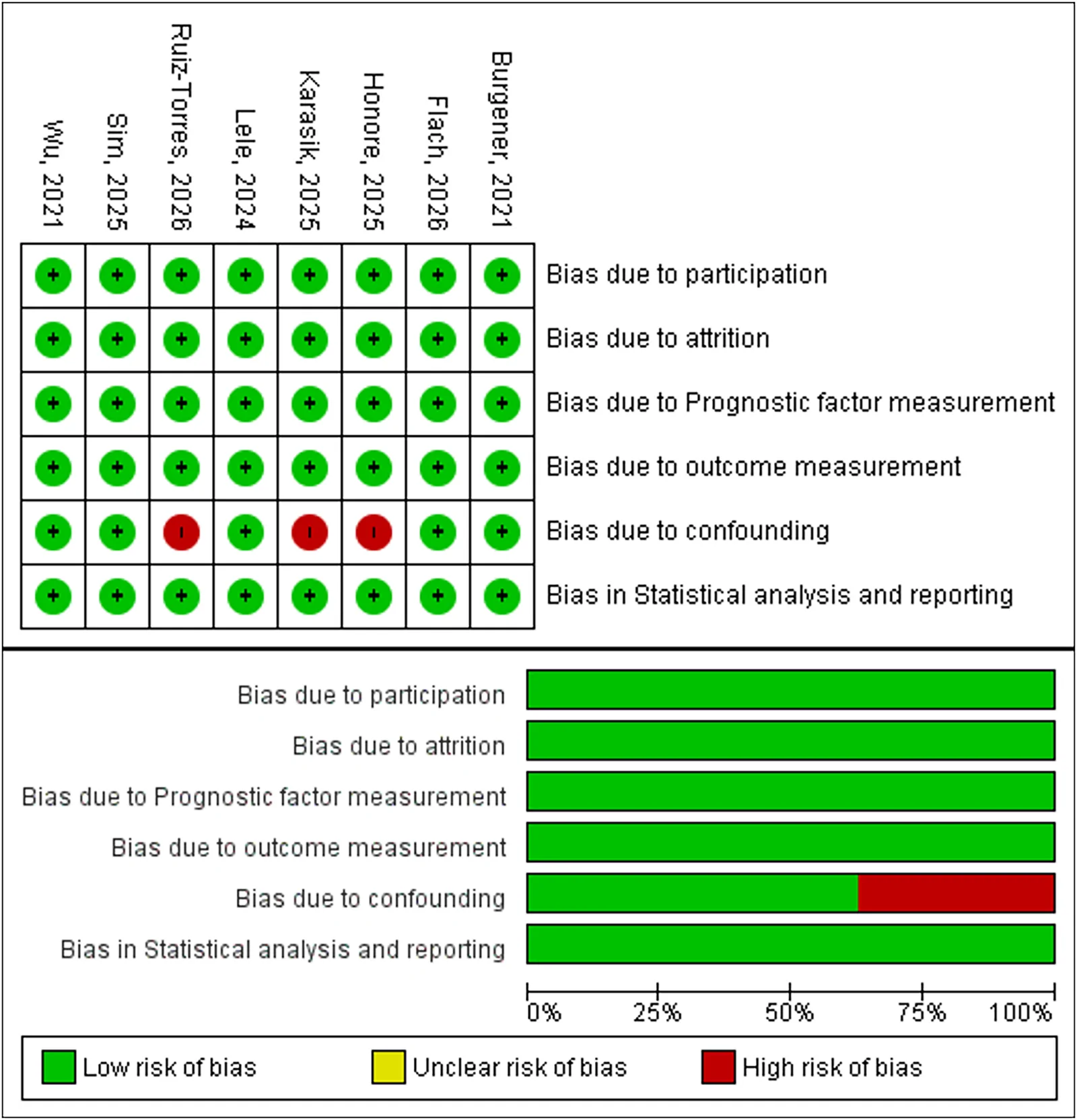
Study quality assessment summary (top) and graph (bottom).

### Post-operative recurrence-free survival

All eight studies reported post-operative ctDNA status and recurrence-free survival outcomes. Meta-analytic aggregation yielded a pooled hazard ratio of 10.07 (95% CI: 5.64–17.99) for the association between detectable post-operative ctDNA and recurrence-free survival (**Figure 3**). This effect magnitude suggests that patients with detectable post-operative ctDNA experienced approximately ten-fold elevated recurrence risk relative to those with undetectable ctDNA. Individual study estimates demonstrated uniform directional concordance, ranging from 5.64 to 22.28, with all confidence intervals excluding the null. Statistical heterogeneity was negligible (I^2^ = 0%; Cochran’s Q p = 0.95), indicating consistency in effect direction and magnitude despite considerable variation in ctDNA detection methodologies, plasma collection timing, and study designs. This homogeneity across diverse technological platforms and institutional settings may suggest that the observed association reflects a robust biological relationship rather than a methodological artifact. The narrow confidence interval and absence of heterogeneity provide some reassurance regarding effect stability, though the relatively small number of contributing studies and the presence of confounding bias in a subset of investigations warrant measured interpretation.

**Figure 3:**
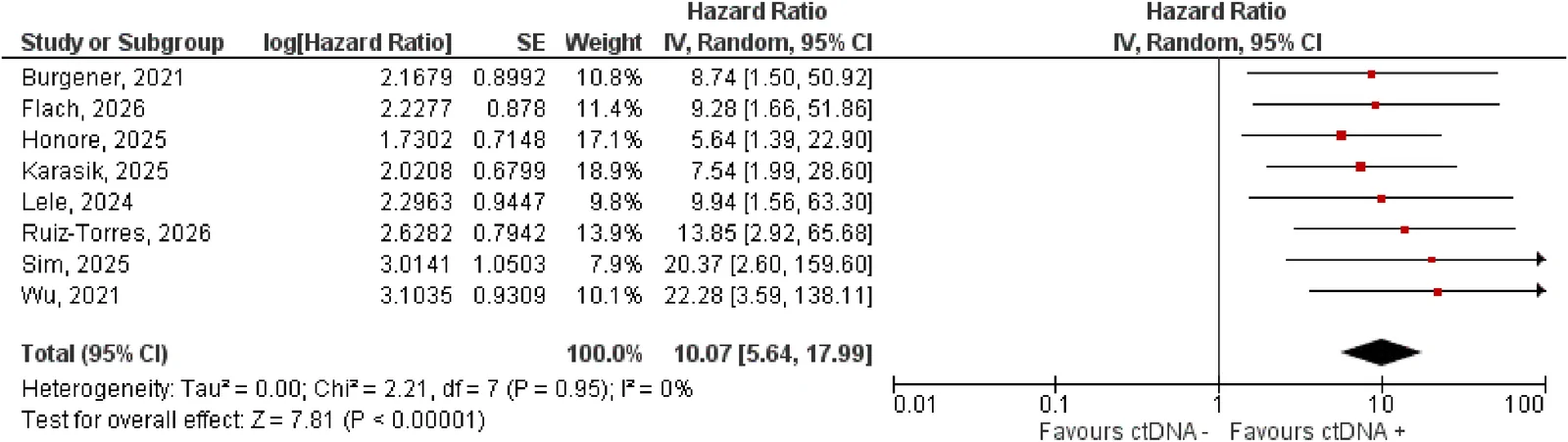
Post operative recurrence free survival and ctDNA.

### Subgroup analysis by cohort composition

Subgroup stratification by patient cohort composition revealed comparable effect estimates across HPV status representation. Three studies (Burgener 2021, Karasik 2025, Wu 2021) enrolled exclusively HPV-negative populations, yielding a pooled hazard ratio of 10.33 (95% CI: 4.12–25.88) for the association between detectable post-operative ctDNA and recurrence-free survival. Five studies (Flach 2026, Honore 2025, Lele 2024, Ruiz-Torres 2026, Sim 2025) enrolled mixed cohorts including both HPV-negative and HPV-positive patients, yielding a pooled hazard ratio of 9.91 (95% CI: 4.69–20.93). Statistical heterogeneity remained negligible within both subgroups (HPV-negative cohorts: I^2^ = 0%, p = 0.63; mixed cohorts: I^2^ = 0%, p = 0.87) (**Figure 4**). The effect estimates between subgroups were comparable in magnitude, with overlapping confidence intervals, suggesting that the observed association between post-operative ctDNA and recurrence-free survival was not substantially influenced by HPV status representation in the study population. Formal testing for subgroup differences indicated no significant heterogeneity between groups (Chi^2^ = 0.00, df = 1, p = 0.94), indicating consistency of the ctDNA-RFS association regardless of cohort composition.

**Figure 4:**
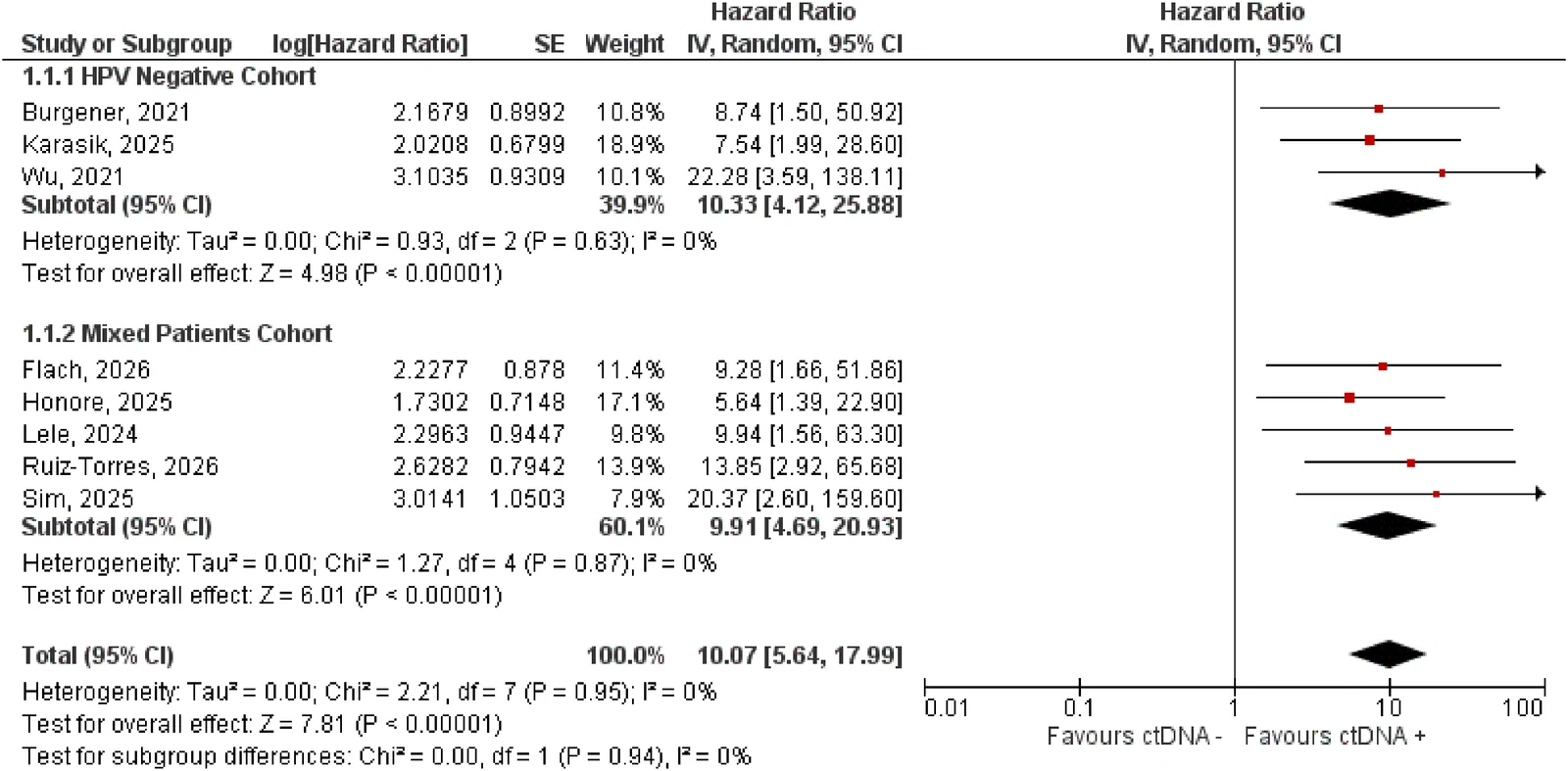
Subgroup stratification by cohort composition.

### Post-operative overall survival: exploratory analysis

Three studies (Honore 2025, Ruiz-Torres 2026, Sim 2025) provided overall survival data, substantially limiting this exploratory analysis. Meta-analytic aggregation yielded a pooled hazard ratio of 8.98 (95% CI: 2.93–27.58) for the association between detectable post-operative ctDNA and overall survival (**Figure 5**). This estimate is comparable in magnitude to the RFS finding, suggesting that ctDNA positivity may influence mortality through mechanisms beyond recurrence alone. Individual study estimates demonstrated consistent directionality, ranging from 6.39 to 18.92, with all confidence intervals excluding the null. Statistical heterogeneity remained negligible (I^2^ = 0%; Cochran’s Q p = 0.72). However, the substantially wider confidence interval relative to the RFS analysis reflects the limited number of contributing studies and reduced statistical precision. These findings warrant particular caution and require validation in larger prospective cohorts. The apparent consistency between RFS and OS associations suggests that recurrence may represent the primary mechanism linking ctDNA positivity to mortality, though alternative pathways remain possible.

**Figure 5:**
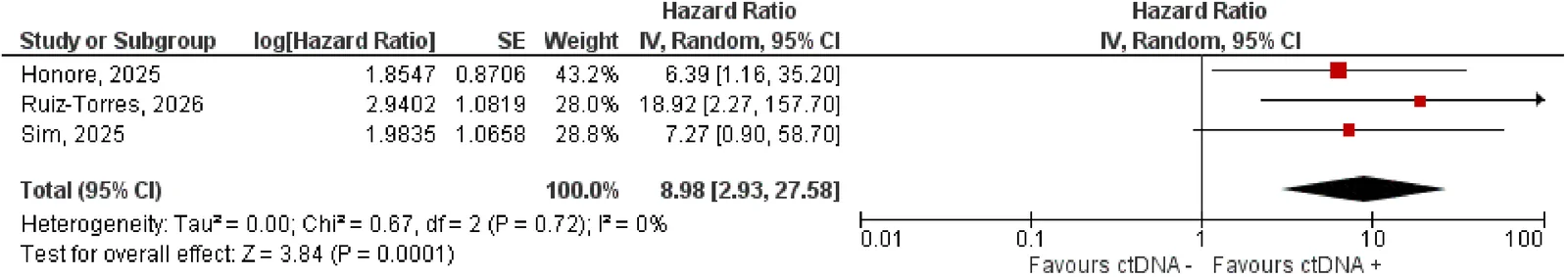
Post-operative overall survival and ctDNA.

### Sensitivity analysis and publication bias assessment

Leave-one-out sensitivity analysis was performed to assess the robustness of the primary meta-analytic estimate. Sequential omission of individual studies yielded pooled hazard ratios ranging from 2.22 (95% CI: 1.61–2.83) when Wu 2021 was excluded to 2.43 (95% CI: 1.79–3.07) when Honore 2025 was excluded. All sensitivity estimates remained statistically significant (p < 0.001), indicating that no single study substantially altered the overall direction or statistical significance of the association. Notably, the range of sensitivity estimates (2.22–2.43) was considerably lower than the primary pooled estimate (10.07), suggesting that the magnitude of the primary effect may reflect the combined influence of all eight studies rather than being driven by individual investigations. This discrepancy between the primary and sensitivity estimates warrants cautious interpretation and underscores the importance of the collective evidence in establishing the observed association (**Figure 6** left).

**Figure 6:**
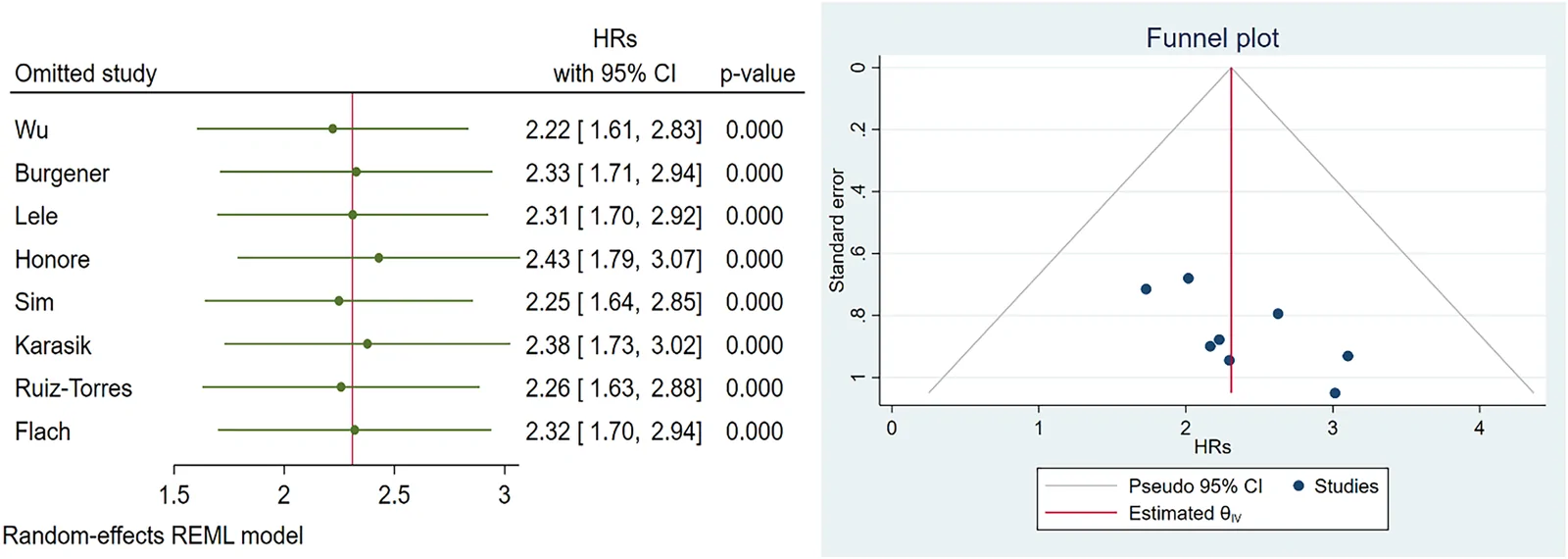
Leave one out sensitivity analysis (Left) and Funnel Plot for publication bias (Right).

Assessment of publication bias was conducted through visual inspection of the funnel plot and formal statistical testing. The funnel plot displayed a generally symmetric distribution of study estimates around the pooled effect, with studies distributed across both sides of the vertical line representing the estimated effect. However, given the limited number of studies (n=8), the statistical power of publication bias tests is inherently constrained. Egger’s regression-based test for small-study effects yielded a beta coefficient of 2.70 (SE = 2.531, z = 1.07, p = 0.286), and Begg’s rank correlation test yielded a Kendall’s score of 14.00 (SE = 8.083, z = 1.61, p = 0.108). While neither test demonstrated statistical significance, the limited sample size restricts the ability to definitively exclude publication bias. The symmetric appearance of the funnel plot provides some visual reassurance, though formal conclusions regarding publication bias should be interpreted with caution given the small number of contributing studies (**Figure 6** right).

## Discussion

This meta-analysis of eight studies yielded a pooled hazard ratio of 10.07 (95% CI: 5.64–17.99) for post-operative ctDNA and recurrence-free survival in HPV-negative HNSCC, with comparable findings for overall survival (HR 8.98). While biologically plausible, these striking effect magnitudes warrant cautious interpretation given substantial methodological limitations.

The findings should not be interpreted as demonstrating that explicitly MRD-focused ctDNA testing is superior to other postoperative ctDNA applications. The non-MRD-focused studies also reported clinically meaningful performance; Wu et al. reported 62.5% sensitivity and 94.7% specificity for postoperative plasma ctDNA, while Lele et al. reported 78% sensitivity and 100% specificity in a small mixed HNSCC cohort (24, 25). The apparent differences between MRD-focused and non-MRD-focused studies may reflect variation in tumour-informed versus tumour-naïve assay design, timing of blood collection, serial testing strategies, treatment pathways, HPV/p16 composition, and endpoint definitions rather than the MRD label itself. Therefore, we provide a narrative comparison and do not perform a pooled sensitivity/specificity or MRD-versus-non-MRD subgroup meta-analysis.

The observed hazard ratios substantially exceed those reported in other solid tumors (colorectal: 2.5–4.0; breast: 2.0–3.5), suggesting potential overestimation. Multiple factors may contribute: heterogeneous detection methodologies with variable sensitivity/specificity; small individual study sample sizes (24–76 patients) susceptible to selection bias; variable plasma collection timing (1–3 days to 3 months post-operatively) affecting ctDNA kinetics; and confounding bias in one-third of studies, with treatment decisions potentially influenced by ctDNA status. Additionally, 62.5% of studies enrolled mixed HPV-positive/negative cohorts, limiting applicability to purely HPV-negative disease. All studies restricted to resectable disease, reducing generalizability to unresectable populations. The small overall sample size (n=325, ~180 HPV-negative) constrains statistical power for meaningful subgroup analyses.

The negligible heterogeneity (I^2^ = 0%) may reflect the limited number of studies rather than true homogeneity. Notably, sensitivity analyses yielded substantially lower estimates (2.22–2.43) than the primary pooled estimate (10.07), suggesting the primary effect depends on collective study influence rather than representing a stable, platform-independent association. This discrepancy raises concerns about robustness and potential unmeasured confounding.

Publication bias testing with n=8 studies has inherent power limitations. While Egger’s (p = 0.286) and Begg’s (p = 0.108) tests were non-significant and the funnel plot appeared symmetric, these findings do not definitively exclude bias. The LIONESS trial, using personalized ctDNA assays in predominantly HPV-positive HNSCC, reported HR ~2.5, substantially lower than the 10.07 observed here, highlighting the need for comparative studies.

While the ctDNA-recurrence association is notable, clinical utility remains unestablished. The heterogeneous detection methodologies preclude recommendations for specific assay platforms. Optimal management of ctDNA-positive patients is undefined, and whether ctDNA-guided interventions improve outcomes is unknown. Large prospective multi-institutional studies with standardized methodologies, enrollment of purely HPV-negative populations, and randomized trials examining ctDNA-guided treatment intensification are needed to establish clinical utility.

## Conclusion

This systematic review and meta-analysis synthesized evidence from eight studies examining the prognostic significance of post-operative ctDNA in HPV-negative HNSCC. While the pooled analysis suggests an association between detectable post-operative ctDNA and recurrence risk, substantial limitations constrain the strength of this evidence. The small number of studies, heterogeneous detection methodologies, inclusion of mixed patient cohorts (62.5% with HPV-positive patients), confounding bias in one-third of investigations, and restriction to resectable disease all limit the generalizability and robustness of the findings. Notably, sensitivity analyses yielded substantially lower effect estimates than the primary analysis, raising concerns about the stability of the pooled result.

The clinical utility of post-operative ctDNA detection for guiding treatment decisions or surveillance strategies in HPV-negative HNSCC remains incompletely established. Until larger, prospective, multi-institutional studies employing standardized methodologies and enrolling purely HPV-negative populations are conducted, post-operative ctDNA detection should be considered an investigational biomarker rather than a clinically validated tool. Randomized controlled trials examining whether ctDNA-guided interventions improve clinical outcomes are essential before recommending routine clinical implementation. Future research should prioritize standardization of detection methodologies and prospective validation in well-characterized HPV-negative HNSCC populations.

## Data Availability

The datasets presented in this study can be found in online repositories. The names of the repository/repositories and accession number(s) can be found in the article/**Supplementary Material**.
