# Supplementary material for: Postoperative ctDNA could potentially spare patients from unnecessary adjuvant therapy in HPV-negative head and neck squamous cell carcinoma: a meta-analysis
Source: Front Oncol. 2026 Oct 16;16:1902328. doi: 10.3389/fonc.2026.1902328 (PMC13719415; doi:10.3389/fonc.2026.1902328)
Supplement: Supplementary file 2 [file Table2.docx]

**Supplementary Appendix**

**Supplementary Data**

**"Prognostic Significance of Post – Operative Circulating Tumor DNA for Recurrence-Free Survival for HPV Negative Squamous Cell Carcinoma of the Head and Neck following Curative Intent Treatment: A Systematic Review and Meta Analysis"**

**Supplementary Table 1**. Detailed Search Strategy

| **Database** | **Keywords / Search String** | **Results** |
| --- | --- | --- |
| **PubMed/MEDLINE** | #1: ("Head and Neck Neoplasms"[MeSH] OR "Head and Neck Squamous Cell Carcinoma"[tiab] OR "HNSCC"[tiab] OR "Oropharyngeal Cancer"[tiab] OR "Laryngeal Cancer"[tiab] OR "Oral Cavity Cancer"[tiab] OR "Hypopharyngeal Cancer"[tiab]) | 8,642 |
|  | #2: ("Papillomavirus Infections"[MeSH:NoExp] OR "Human Papillomavirus"[MeSH] OR "HPV"[tiab] OR "HPV-negative"[tiab] OR "HPV-independent"[tiab] OR "HPV negative"[tiab] OR "HPV independent"[tiab]) | 31,245 |
|  | #3: ("Survival Analysis"[MeSH] OR "Overall Survival"[tiab] OR "Disease-Free Survival"[tiab] OR "Progression-Free Survival"[tiab] OR "Recurrence"[MeSH] OR "Prognosis"[MeSH] OR "Hazard Ratio"[tiab] OR "Kaplan-Meier"[tiab]) | 287,654 |
|  | #1 AND NOT #2 AND #3 | 1,452 |
| **Web of Science** | ("Head and Neck Squamous Cell Carcinoma" OR "HNSCC" OR "Oropharyngeal Cancer" OR "Laryngeal Cancer" OR "Oral Cavity Cancer" OR "Hypopharyngeal Cancer") AND NOT ("HPV-positive" OR "Human Papillomavirus positive") AND ("Overall Survival" OR "Disease-Free Survival" OR "Progression-Free Survival" OR "Recurrence" OR "Prognosis" OR "Hazard Ratio") | 1,215 |
| **Scopus** | TITLE-ABS-KEY (("Head and Neck Squamous Cell Carcinoma" OR "HNSCC" OR "Oropharyngeal Cancer" OR "Laryngeal Cancer" OR "Oral Cavity Cancer" OR "Hypopharyngeal Cancer") AND ("HPV-negative" OR "HPV-independent" OR "HPV negative" OR "HPV independent" OR NOT "HPV-positive") AND ("Overall Survival" OR "Disease-Free Survival" OR "Progression-Free Survival" OR "Recurrence" OR "Prognosis" OR "Hazard Ratio")) | 1,640 |
| **CINAHL** | (MH "Head and Neck Neoplasms") OR TI ("Head and Neck Squamous Cell Carcinoma" OR "HNSCC" OR "Oropharyngeal Cancer" OR "Laryngeal Cancer" OR "Oral Cavity Cancer") AND TI ("HPV-negative" OR "HPV-independent" OR "HPV negative") AND TI ("Overall Survival" OR "Disease-Free Survival" OR "Progression-Free Survival" OR "Recurrence" OR "Prognosis" OR "Hazard Ratio") | 487 |
| **Cochrane Central Register of Controlled Trials (CENTRAL)** | ("Head and Neck Squamous Cell Carcinoma" OR "HNSCC" OR "Oropharyngeal Cancer" OR "Laryngeal Cancer" OR "Oral Cavity Cancer") AND ("HPV-negative" OR "HPV-independent" OR "HPV negative") AND ("Overall Survival" OR "Disease-Free Survival" OR "Progression-Free Survival" OR "Recurrence" OR "Prognosis") | 142 |
| **ClinicalTrials.org** | Condition: Head and Neck Cancer OR Oropharyngeal Cancer OR Laryngeal Cancer OR Oral Cancer; Intervention: Chemotherapy OR Radiotherapy OR Immunotherapy OR Surgery; Other Terms: HPV-negative OR HPV-independent; Status: Recruiting, Active, Completed | 156 |
